# A Continuous Cell Separation and Collection Approach on a Microfilter and Negative Dielectrophoresis Combined Chip

**DOI:** 10.3390/mi11121037

**Published:** 2020-11-26

**Authors:** Qiong Wang, Xiaoling Zhang, Danfen Yin, Jinan Deng, Jun Yang, Ning Hu

**Affiliations:** 1 Key Laboratory of Biorheological Science and Technology, Ministry of Education, Bioengineering College, Chongqing University, Chongqing 400030, China; wangqiong@cqu.edu.cn (Q.W.); yindf@cqu.edu.cn (D.Y.); biojdeng@cqu.edu.cn (J.D.); bioyangjun@cqu.edu.cn (J.Y.); 2 School of Health and Aging Service, Chongqing City Management College, Chongqing 401331, China

**Keywords:** particle/cell separation, micropost array railing, dielectrophoresis, microfilter

## Abstract

Cell separation plays an important role in the fields of analytical chemistry and biomedicine. To solve the blockage problem and improve the separation throughput in the traditional microstructure filtration-based separation approach, a continuous cell separation and collection approach via micropost array railing on a microfilter and negative dielectrophoresis combined chip is proposed. By tilting the micropost array at a certain angle, microparticles or cells enter the collection area under micropost array railing. The effects of the inclination angle of the micropost array and the electrode distance on the microparticle collection efficiency were investigated. Based on the optimized microfluidic chip structure, 37- and 16.3-μm particles were collected with 85% and 89% efficiencies, respectively. Additionally, algal cells were separated and collected by using the optimized microchip. The chip also had good separation and collection effects on biological samples, which effectively solved the blockage problem and improved the separation throughput, laying a foundation for subsequent microstructure filtration separation-based research and application.

## 1. Introduction

Microfluidic chip technology [1,2] is a new technology that integrates various microchannels and corresponding driving, reaction, detection and other functional modules to realize sampling, dilution, reaction, separation, detection and other operations involved in the analysis process in the fields of biology, chemistry and medicine [3]. This technology can realize the function of a complete biochemical laboratory on a small chip, so it is also popularly known as a “lab-on-a-chip” or micro total analysis system (μTAS). At the same time, due to its small size, low sample consumption, fast reaction and analysis speeds and high integration, it is capable of high throughput, large sample parallelization and fast synchronous analysis [4]. The miniaturization and integration of microfluidic chips make them extremely portable and able to conduct experiments wherever needed. These advantages make microfluidic chip technology widely used in chemical industry process control, environmental monitoring, forensic identification, biological analysis [5], clinical testing [6], food safety [7,8] and other fields.

The separation of biological samples is an important branch in the modern biomedical research field. Cell separation plays an important role in the fields of analytical chemistry and biomedicine [8]. In practical applications, such as clinical examination, target cells usually need to be separated and enriched from samples before subsequent analysis and diagnosis. The traditional biological sample sorting method can be divided into the gravity effect, electromagnetic labeling, antigen and antibody labeling, etc. In general, the above separation methods require a large amount of biological sample; the purity of the samples is relatively low and susceptible to contamination; and the sorting process is complex, which often requires complicated multistep operations. Therefore, the traditional separation method used in clinical testing is inefficient, which brings inconvenience to the relevant analysis and testing. Compared with the traditional separation method, microfluidic chips have natural advantages in biological sample separation and have attracted extensive attention from many researchers [9,10,11,12]. Microfluidic separation of cells and particles is also a key application of microfluidic chips [13].

Generally, cell separation methods based on microfluidic chips can be divided into two categories: passive [14,15,16] and active [17,18,19,20]. The passive methods have no requirements for the properties of the cells, and the cells do not need to be prelabeled; thus, the survival rate after the separation is high, and the experimental operation is simple [21]. However, the microfluidic chip used in passive separation has higher requirements on the microstructures integrated on the chip, so complex and well-defined microstructures need to be designed to ensure better cell separation results [22]. For example, in the micropost filtration and separation method [23,24,25,26], the gap between microposts directly determines the size of the filtered cells, which has an important influence on the separation efficiency, putting forward a higher requirement for the chip processing accuracy. Meanwhile, in passive methods, the microstructure is often blocked by the samples, which affects the separation of subsequent samples and limits the separation throughput. Otherwise, a parallel structure needs to be integrated on the chip to improve its separation throughput, but this increases the chip complexity and the design and processing costs. The microfluidic chip based on active separation has a relatively simple structure and a higher separation efficiency than that based on passive separation. However, active separation methods usually have certain requirements for target cells (such as an obvious difference in size and other parameters), and most of them require pretreatment, such as labeling of samples, which has a certain impact on subsequent analysis, detection and cell activity. Thus, passive and active methods have sometimes been combined to obtain higher separation efficiency, which can overcome some disadvantages of a single method.

**Figure 1:**
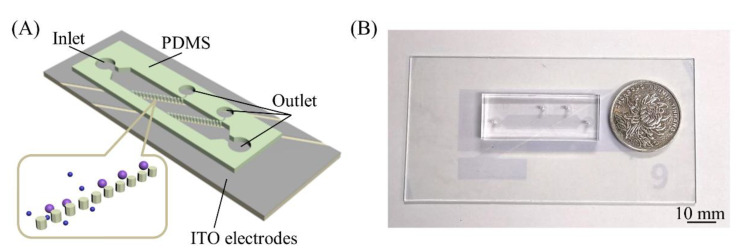
(**A**) Conceptual illustrations of the overall device and enlarged schematic; and (**B**) picture of the device.

To solve the blockage problem in traditional microstructure filtration separation, we proposed a microfluidic chip by integrating microelectrodes into the microfilter structure [27]. We previously introduced negative dielectrophoresis (nDEP) into the microfilter chip and demonstrated that this design can avoid the blockage problem. Dielectrophoresis, which does not require labeling of cells, is simple and useful for cell or particle separation. nDEP can drive cells to the low electric field intensity area, thus avoiding cell blockage in the region of the filter hole. Although the blockage problem of the traditional microstructure filtration method is solved and high-throughput continuous separation has been achieved [27], when the number of particles/cells in the chip increases to a certain extent, the filter hole will eventually be blocked by particles/cells. To better solve the blockage problem and improve the chip throughput, we improved the chip by tilting the micropost array at a certain angle to form a particle/cell track; thus, particles/cells would enter the collection area under micropost array railing (μPAR) [28]. The microposts arrayed at an angle with respect to the flow direction serve as a railing system for directing microparticles or cells into different fluidic streams [28]. Figure 1 depicts the concept of the microfluidic device. Cells or particles are introduced into the flow chamber by the flows. The negative DEP force acting on small cells is smaller than the fluid flow, so they will pass through the filter holes under the fluid flow. Big cells cannot pass through the filter holes and will rail along the tracks into the collection area.

## 2. Materials and Methods 

### 2.1. Theory and Simulations

**Figure 2:**
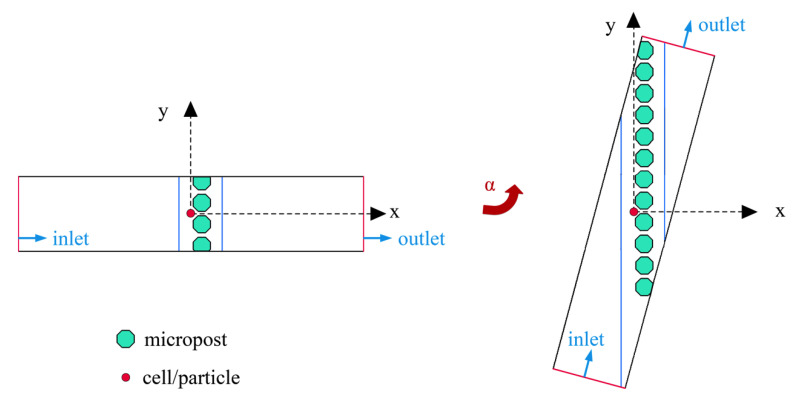
Geometry for simulating the particle trajectories.

To predict the trajectories of the cells or particles in the microchannel, an arbitrary Lagrangian–Eulerian (ALE) method-based numerical model was used to simulate the electrokinetic motions of spherical particles. This numerical method was developed in reference to the study by Qian et al. [29]. To simplify the simulation, we focus on a part of the chip, the microarray area (see Figure 2). A circular particle is suspended in an incompressible Newtonian fluid confined in a rectangle of length *L* and width *W*. The particle is initially located before a micropost array. The rectangle (microchannel) can be rotated around the center of the particle to form a different angle (α) between the microarray and the normal direction of the inputted flow. The number of microposts varies with the rotation angle, and the microposts fill the entire microchannel. An AC electric field is applied between the two electrodes (blue lines in Figure 2) across the microarray to induce negative DEP to avoid the blockage problem. To investigate the influence of the inclination angle of the micropost array on the collection efficiency, the distances between the particle, microposts and electrodes are kept the same; only the microchannel orientation is changed from 0 to α (15°, 30°, 45°, 60° and 75°). 

Specifically, the distribution of the electric potential in the fluid and the particle is governed by Gauss’s law [29], given by

(1) $$\nabla \cdot \left({\tilde{\varepsilon }}_{f}\nabla {\tilde{\phi }}_{f}\right)=0$$

and

(2) $$\nabla \cdot \left({\tilde{\varepsilon }}_{p}\nabla {\tilde{\phi }}_{p}\right)=0$$

where ${\tilde{\varepsilon }}_{f}=\varepsilon_{f}-j\sigma_{f}/\omega$ and ${\tilde{\varepsilon }}_{p}=\varepsilon_{p}-j\sigma_{p}/\omega$ are, respectively, the complex permittivity of the fluid and particle. *ε_f_* (*ε_p_*) and *σ_f_* (*σ_p_*) are the permittivity and conductivity of the fluid (the particle), respectively. *ω* = 2π*f* is the AC electric field’s angular frequency (*f* is the AC field frequency), $j=\sqrt{-1}$ is the imaginary vector. The superscript “*~*” represents complex variables.

An electric potential is applied between the electrodes (blue lines) to generate an AC electric field and is given by

(3) $$\tilde{\phi }=\phi_{0}$$

and

(4) $$\tilde{\phi }=0$$

The electric potential and the normal component of the electric displacement are both continuous at the interface between the fluid and particle, and electric insulation is applied at all the other boundaries.

Due to the extremely small Reynolds number of the fluid flow, the fluid flow is governed by the Stokes equations and the continuity equation, given by

(5) $$\rho_{f}\frac{\partial u}{\partial t}=-\nabla p+\eta \nabla^{2}u$$

(6) ∇·u=0

where *ρ_f_* is the fluid density, **u** is the fluid velocity vector and *p* is the fluid pressure. The fluid velocity at the inlet is set at *U*_0_ for all of the velocity field simulations. A normal flow with no external pressure gradient (i.e., *p* = 0) is specified at the outlet, and the other boundaries are no-slip.

The time-averaged DEP force, **F***_DEP_*, and the hydrodynamic force, **F***_H_*, acting on the particle are obtained by integrating the time-averaged Maxwell stress tensor, **T***_M_*, and the hydrodynamic stress tensor, **T***_H_*, over the surface of the particle, given by

(7) $$F_{DEP}=\int^{\;}T_{M}\cdot ndS=\int^{\;}\frac{\varepsilon_{f}}{4}\left[\left({\tilde{E}}_{f}{\tilde{E}}_{f}^{\prime }+{\tilde{E}}_{f}^{\prime }{\tilde{E}}_{f}\right)-{\left|{\tilde{E}}_{f}\right|}^{2}I\right]\cdot ndS$$

and

(8) $$F_{H}=\int^{\;}T_{H}\cdot ndS=\int^{\;}\left[-pI+\eta \left(\nabla u+{\left(\nabla u\right)}^{T}\right)\right]\cdot ndS$$

where ${\tilde{E}}_{f}$ and ${\tilde{E}}_{f}^{\prime }$ are the electric field strength and the complex conjugate of ${\tilde{E}}_{f}$.

The fluid velocity on the particle surface is expressed as

(9) $$u_{i}=U_{pi}+\omega_{pi}\times \left(x_{si}-x_{pi}\right)$$

The translational velocity ($U_{pi}$) and rotational velocity ($\omega_{pi}$) of the particle are determined by

(10) $$m_{pi}\frac{dU_{pi}}{dt}=F_{Hi}+F_{DEPi}$$

(11) $$I_{pi}\frac{d\omega_{pi}}{dt}=\int^{\;}\left(x_{si}-x_{pi}\right)\times \left[\left(T_{H}+T_{M}\right)\cdot n\right]dS_{i}$$

where $m_{pi}$ and $I_{pi}$ are the mass and moment of inertia of the particle, respectively. The position and orientation of the particle can be obtained by

(12) $$\frac{dx_{pi}}{dt}=U_{pi}$$

(13) $$\frac{d\theta_{pi}}{dt}=\omega_{pi}$$

All the governing equations are normalized to simplify the calculation in reference to the study by Qian et al. [29], and the mathematical model is numerically solved by COMSOL Multiphysics 3.5a (www.comsol.com).

### 2.2. Device Fabrication

The proposed microfluidic device is composed of a polydimethylsiloxane (PDMS) microstructure layer and an indium-tin-oxide (ITO) microelectrode layer. The PDMS microstructure layer was fabricated by using the standard soft lithography technique. After fabricating a 50-μm thick positive mold on a 3-inch silicon wafer (ePAK, Austin, TX, USA) by using SU-8 3050 (Microchem, Westborough, MA, USA), 20 g PDMS (Dow Corning, Midland, MI, USA) and a curing agent in a ratio of 10:1 were used to replicate the mold to form the microfilter and collection structure, as shown in Figure 1A. Transparent ITO glass was used to fabricate the microelectrode structure. The fabrication process can be found in a previous publication [27]. A PDMS replica was bonded onto the ITO glass via O_2_-plasma activation (PDC-MG, Chengdu, China). The microfluidic device had four reservoirs with a diameter of 4 mm and a long microchannel with a varied length, a width of 6 mm and a depth of 50 μm. The device consisted of a series of octagonal microposts arrayed at an angle in the channel, and the gaps between microposts were 25 (the first stage) and 14 μm (the second stage). The distance between the two ITO electrodes (d) was wider than the microposts, varying from 300 to 800 μm.

### 2.3. Experimental Solution Preparation

Nonbiological samples and biological samples were used to verify the designed microfluidic device. Nonbiological samples were mixtures of polystyrene particles of different sizes: 37-, 16.3- and 9.7-μm diameters. Then, 1 mM phosphate-buffered saline (PBS) buffer (10% PBS buffer, 0.17 S/m conductivity) [30,31] was used to adjust the particle concentration to 10^4^–10^5^/mL. To prevent adhesion of particles to particles and to the microchannel walls, Tween 20 (Bomeibio, China) at a 0.1% *v/v* concentration was added into the suspending medium.

A mixture of *Haematococcus pluvialis* (FACHB, Wuhan, China) and *Bracteacoccus engadinensis* (FACHB, Wuhan, China) was selected for biological samples. Both algae were cultured in Blue-Green medium (BG11, FACHB, Wuhan, China). Before the experiments, the algae mixture was adjusted to a concentration of 10^4^–10^5^/mL in the culture medium. It should be noted that, due to the high density, algae easily sank to the chip bottom. Therefore, 0.4 g/mL d-sorbitol was added to suspend the algae.

### 2.4. Experimental Setup, Visualization and Data Analysis

Prior to the experiments, the outlet reservoir of the microfluidic device was connected to a peristaltic pump through a pipe. The peristaltic pump created a negative pressure to drive the particles/cells and buffer in the inlet reservoir to flow into the microchannel. A signal generator (SDG1020, Siglent, Solon, OH, USA) was connected to a voltage amplifier (ATA-2042, Agitek, Xi’an, China) and then connected to the ITO electrodes through conductive adhesive tape to provide the required sinusoidal signals. A camera (D70, Canon, Tokyo, Japan) and an inverted microscope (IX73, Olympus, Tokyo, Japan) were used to observe and record the entire experimental process. Before the experiments, the microchip was rinsed with 1 mM PBS buffer for 5 min. Then, 100-μL samples were loaded at the inlet reservoir. Under negative pressure, the samples could be driven towards the outlet. AC signals of 10 kHz for particles and 8 kHz for algae cells were loaded on the ITO electrodes to achieve separation and collection manipulation, and the amplitudes of the AC signals could be adjusted according to the size of the particles or algae cells.

As the absolute number of particles or cells collected in a single experiment is not stable, to evaluate the collection efficiency of each individual experiment, the collection efficiency (*CE*) of the microchip was calculated as

(14) $$CE=\frac{N_{c}}{N_{c}+N_{f}}$$

where *N_c_* is the number of particles or cells in the collection area and *N_f_* is the number of particles or cells in the filter area.

## 3. Results and Discussion

### 3.1. Simulation Results

The suspending medium and particles used in the simulation and experiments are 10% PBS buffer and polystyrene particles. The fluid density *ρ_f_* is 1 × 10^3^ kg/m^3^ and the fluid viscosity *µ* is 1 × 10^−3^ Pa·s. The particle density was set to be the same as that of the fluid, so the gravity effect can be neglected. The conductivity and permittivity of the fluid and the particle are *σ_f_* = 0.17 S/m and *ε_f_* = 80*ε*_0_ and *σ_p_* = 4.0 × 10^−4^ S/m and *ε_p_* = 2.6*ε*_0_, respectively, where *ε*_0_ is the absolute permittivity of vacuum. The applied potential *ϕ*_0_ and frequency *f* are 30 V and 10 kHz, respectively. The diameters of the particles used in the simulations are 20 μm and 10 μm. The radius of the smaller particle, *a* = 5 μm, is set as the characteristic length, the length of the microchannel is *L* = 240*a*, the width is *W* = 52*a* and the distance between the electrodes is 30*a*.

**Figure 3:**
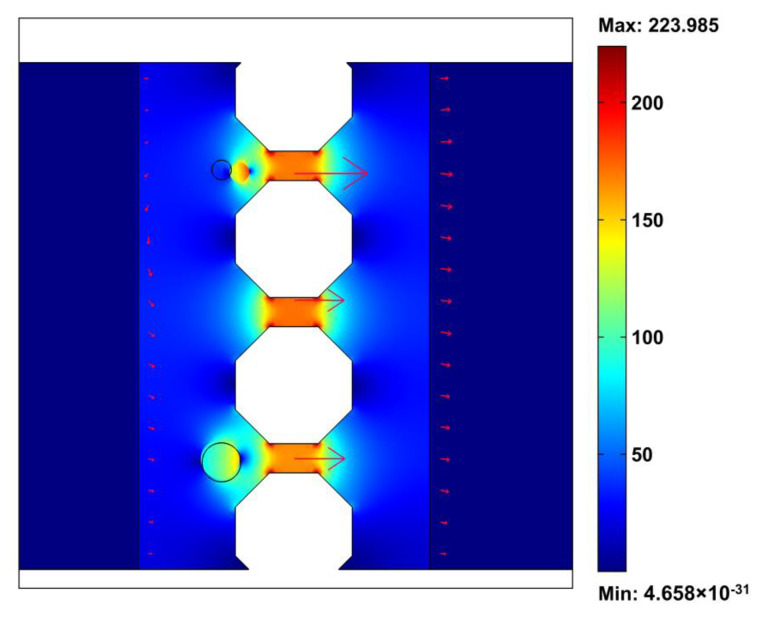
Distribution of the electric field and the movement of different size particles on a microfilter and negative dielectrophoresis combined chip. Arrows represent the direction of the flow field.

**Figure 4:**
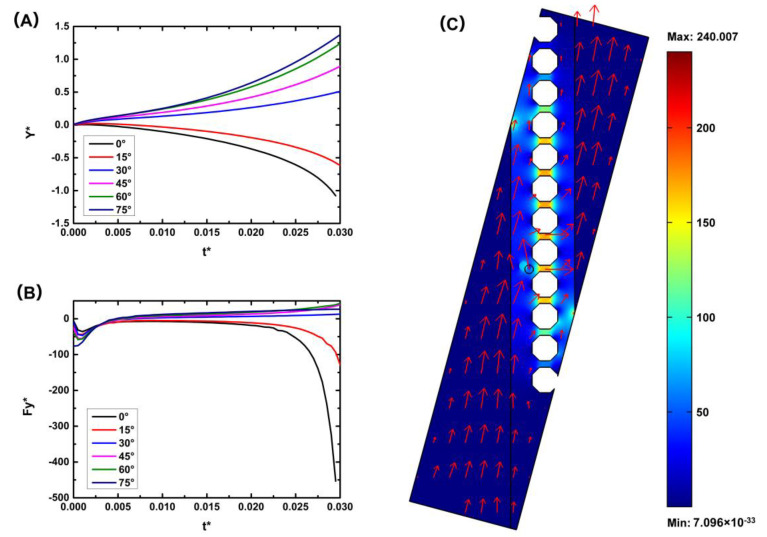
Simulation results of the influence of the inclination angle of the microfilter array on the collection efficiency: (**A**) Y* (the normalized displacement of *y* direction); (**B**) Fy* (the normalized y-component of the total force acting on the particle); and (**C**) electric field and flow field when α = 75°.

We assume that a particle is located near the micropost array. When the micropost array is perpendicular to the direction of the inputted fluid flow (*α* = 0), this is the traditional geometry configuration of the microfilter device. When the nDEP force is small, the particle moves forward under the effect of the fluid drag force and can pass through the microfilters or be stuck at the filters. When the nDEP force is large enough, the particle moves towards the low electric field area and away from the microfilters or stops before the micropost array, as shown in Figure 3. The distributions of the electric field strength and flow velocity are also shown in Figure 3. A nonuniform electric field is generated in the microchannels, and the electric field is focused near the microfilter area. The direction of the flow velocity is perpendicular to the microfilter array, and, basically, there is no component of the fluid force towards the collection area. Negative DEP can solve the blockage problem of the microfilter device, consistent with prior experiments [27], but the particle can hardly move to the collection area. Therefore, we improved the chip by tilting the micropost array at a certain angle to form a cell track; thus, particles would enter the collection area under micropost array railing (μPAR). We investigated the influence of the inclination angle of the microfilter array (*α*) on the collection rate. Dynamic simulations were performed for the microsystems with values of *α* varying 0°–75° (Figure 4). Figure 4C shows the simulated electric field strength and flow velocity across a device model for the 75° design. The inclined microfilter array changes the direction of the fluid velocity such that the particle has a tendency to move in the *y* direction, that is, in the direction of the collection area. The simulation results also show that with an increase in the inclination angle of the micropost array (*α*), the displacement along the microarray line (*y* direction) at the same time is larger. That is, the speed of the particle railing is faster, and the corresponding collection efficiency is higher.

### 3.2. Particle Collection Experiment

#### 3.2.1. Influence of the Inclination Angle of the Micropost Array (α) on the Collection Efficiency

**Figure 5:**
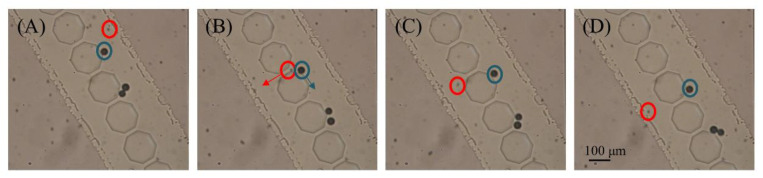
Sequential images of particle separation and collection in the first stage (**A**–**D**). The 37-μm particles (in the blue circle) rail along the tracks into the collection area and the 16.3-μm particles (in the red circle) pass through the filter holes.

In previous simulations, the inclination angle of the micropost array had an important influence on the flow of particles on the microfluidic chip. To further reduce the blockage of the filter hole by particles and improve the collection efficiency of particles, the influence of the inclination angle on the collection efficiency of particles was analyzed. Here, we designed four different angles (30°, 45°, 60° and 75°) to study the influence of the inclination angle on the particle collection efficiency. As the 37-μm particles only existed in the first stage, they could only be distributed near the first filtration stage or collection area. The 16.3-μm particles were distributed near the first and second filtration stages and in the collection areas and the 9.7-μm particles were distributed throughout the chip. The 37-μm particles have a small distribution area and fewer influencing factors, making them suitable for evaluating the influence of the inclination angle on the particle collection efficiency. Therefore, 37-μm particles were selected as the experimental observation object. The electrode distance was 500 μm in the first stage and 300 μm in the second stage. The output voltage signal was fixed at 10 kHz and 100 V_p-p_. Each experiment was repeated three times. Figure 5 shows a continuous particle separation and collection in the first stage (the complete process is recorded in Video S1 and the separation and collection process in the second stage is recorded in Video S2).

**Figure 6:**
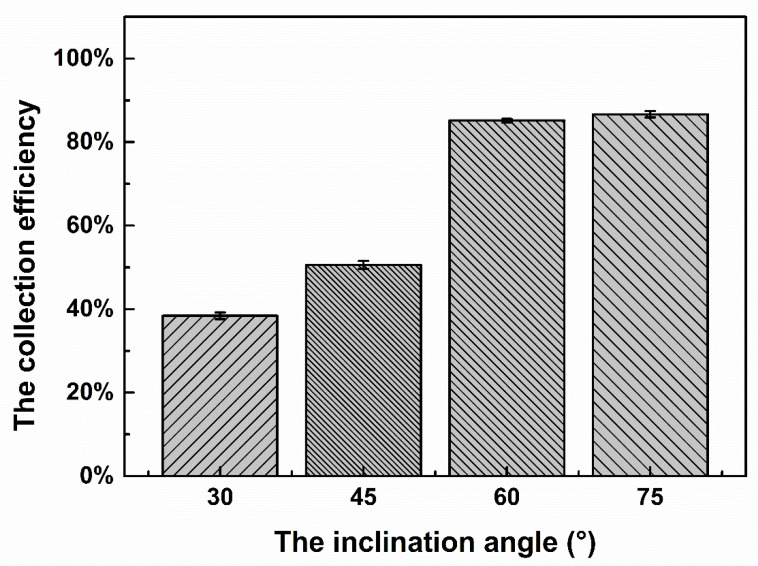
Effect of the inclination angle on the collection efficiency of 37-μm particles.

Inclination of the micropost array causes a change in the direction of the fluid near the microfilter and the direction of the dielectric electrophoretic force on the particles near the filter array. Therefore, the inclination angle of the micropost array directly affects the proportion of particles in the collection area. Figure 6 shows the collection efficiency (the proportion of particles in the collection area) at the four inclination angles. As shown in the figure, as the inclination angle increases from 30° to 60°, the proportion of particles in the collection area increases from 38% to 85%, indicating that the inclination angle affects the particle collection results, and it is more likely for particles to enter the collection area with an increase in the inclination angle. However, when the inclination angle increases from 60° to 75°, the proportion in the collection area only increases by 2%, indicating that an increase in the inclination angle above a certain angle has no great influence on the collection efficiency, and the collection efficiency of particles has reached close to a maximum (~90%). The observation that some particles remain near the microfilter array may be due to the adhesion between the particle and the chip or between particles. With an increase in the inclination angle, the length of the microchip also increases (comparing the 75° inclination angle chip with the other three inclination angle chips), and it is harder to observe the movement of particles. Considering that there is only a 2% difference between the 75° inclination angle chip and the 60° inclination angle chip, we finally chose 60° as the inclination angle of the microfilter array.

#### 3.2.2. Influence of the Electrode Distance on the Collection Efficiency

**Figure 7:**
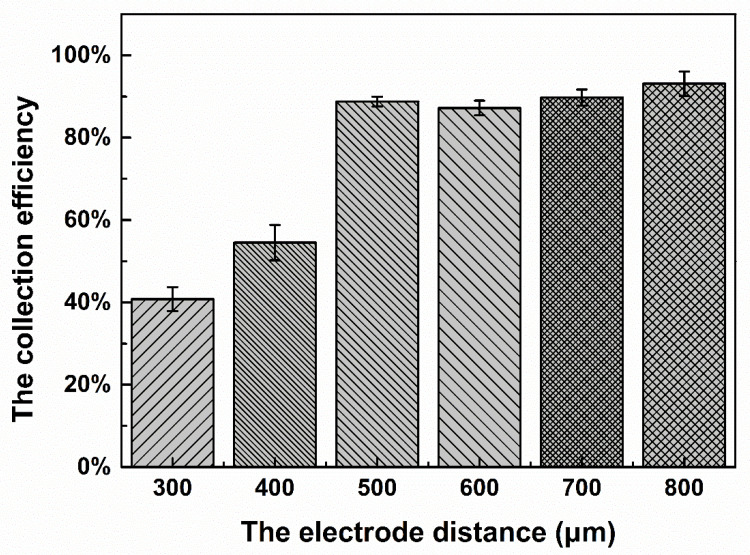
Effect of the electrode distance on the collection efficiency of 16.3-μm particles.

After setting the inclination angle, the experimental conditions need to be further optimized. For a certain inputted fluid velocity, the applied electric field is an important experimental condition. According to *E* = *V*/*d*, when the voltage is constant, the smaller the electrode distance is, the stronger the electric field intensity will be. To investigate the influence of the electrode distance on the collection efficiency, the electrode distance of the second stage was fixed at 300 μm, and the electrode distance of the first stage was changed. The distributions of 16.3-μm particles at all stages were calculated to evaluate the collection efficiency. The distribution of particles was calculated as the number of particles of a particular size collected at a certain stage/the total number of particles of that size collected. The electrode distance of the first stage ranged from 300 to 800 μm, and each experiment was repeated three times. The relationship between the electrode distance and the 16.3-μm particle collection efficiency is shown in Figure 7. As shown in Figure 7, as the electrode distance increased from 300 to 500 μm, the collection efficiency of 16.3-μm particles increased greatly from 41% to 89%. The collection efficiency of 9.7-μm particles was calculated and was maintained at approximately 90%, indicating that the change in the electrode distance had no influence on the collection efficiency of 9.7-μm particles, while the collection efficiency of 16.3-μm particles had a strong relationship with the electrode distance. The reasons for this are as follows: First, the size of the 9.7-μm particles is small, and the dielectrophoretic force is always smaller than the fluid force; thus, the change in the dielectrophoretic force caused by the change in the electrode distance has little influence on the trajectory of the 9.7-μm particles. Second, at the same voltage, the dielectrophoretic force generated for a distance of 300 μm is greater than that generated for a distance of 500 μm, resulting in a stronger dielectrophoretic force for 16.3-μm particles moving towards the collection area in the first stage and a decrease in the number of particles collected in the second stage. Therefore, as the electrode distance increased, the dielectrophoretic force decreased, fewer 16.3-μm particles were collected in the first stage, while more 16.3-μm particles were collected in the second stage, and the collection efficiency was improved. The collection efficiency of 16.3-μm particles exhibits little change from 500 to 800 μm and is approximately 90%. This indicates that, when the electrode distance is greater than 500 μm, the collection efficiency of 16.3-μm particles does not change significantly with a change in the electrode distance, and the maximum collection efficiency of 16.3-μm particles is approximately 90%. On the other hand, an increase in the electrode distance results in a decrease in the dielectrophoretic force on 37-μm particles, resulting in a decrease in the number of 37-μm particles moving towards the collection area, an increase in the number of particles remaining near the filter hole, and an increase in the probability of the filter hole being blocked. Thus, an electrode distance of 500 μm was selected after considering the collection efficiency and quantity.

#### 3.2.3. Optimized Results

**Figure 8:**
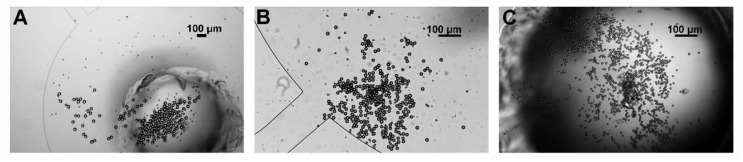
Particle collection results: (**A**) particles in the first-stage collection area; (**B**) particles in the second-stage collection area; and (**C**) particles in the outlet reservoir.

The optimized chip structure was used for separation and collection experiments, and the results are shown in Figure 8. Figure 8A shows the collection results of the first stage, most of which are 37-μm particles. However, due to the inclination of the filtration array, when 37-μm particles move towards the collection area, some 16.3- and 9.7-μm particles also move towards the collection area along the filtration array, resulting in some 16.3- and 9.7-μm particles appearing in the first-stage collection area. Figure 8B shows the collection result of the second stage. There are 16.3-μm particles and a small number of 9.7-μm particles in the collection area, and the particles are slightly clustered and overlapped. Figure 8C shows the results of the experiment at the outlet. There are many particles, all of which are 9.7-μm particles. According to statistics, 37-μm particles were distributed in the first-stage collection area. The collection efficiency of 16.3-μm particles reached 90% in the second stage and only 10% in the first stage. Ninety-three percent of 9.7-μm particles were exported to the outlet reservoir. The microfluidic device has a good separation effect on the three kinds of particles and can also collect the separated particles, which is conducive to reducing the risk of blockage, extending the chip working time and increasing the chip throughput.

### 3.3. Algae Cell Collection Experiment

**Figure 9:**
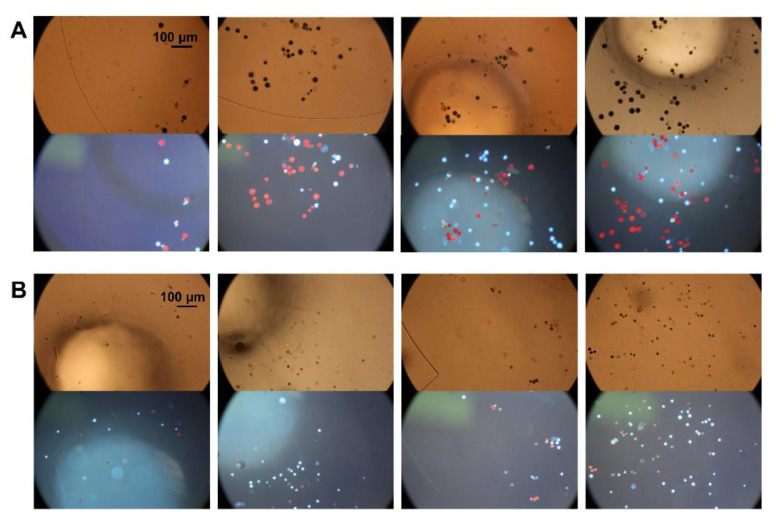
Algae cell collection results: (**A**) algae cells in the first-stage collection area; and (**B**) algae cells in the second-stage collection area.

**Figure 10:**
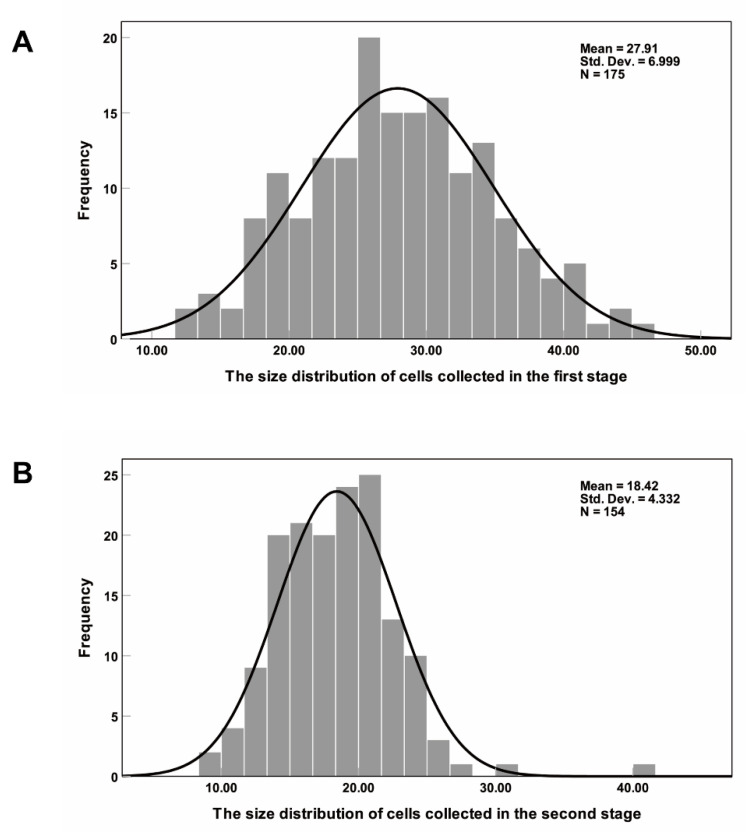
Cell size distribution in the collection areas: (**A**) size distribution of cells collected in the first stage; and (**B**) size distribution of cells collected in the second stage.

The algal cells have spontaneous fluorescence and can be seen clearly under a fluorescence microscope, with living and dead cells showing different colors. Figure S1 shows the morphology of algal cells under: bright field (A); green light (B); blue light (C); and ultraviolet light (D) excitation. Under the bright field, it can be judged that those cells with an incomplete cell structure are dead cells, while cell states cannot be distinguished when the cells have a complete cell structure. Living cells are red under all three types of light, while dead cells are yellow under green and blue light and white under ultraviolet light. The contrast between dead cells and living cells is the most obvious in Figure S1D. The living cells’ intracellular materials are red, and the cell wall structure is also clearly visible. The dead cells have no complete membrane structure, and they are all white within the range of the cell wall. Under bright field, the color of some dead cells is too shallow, and it is easy to ignore these cells when counting, causing statistical error. Under ultraviolet light, every cell can be easily observed, which is convenient for counting and statistics. Therefore, in this experiment, ultraviolet light was adopted as the excitation light source, under which living cells are red and dead cells are white. The collection results are shown in Figure 9. Figure 9A shows the first-stage collection results and Figure 9B shows the second-stage collection results. By comparing the cell sizes in the two figures, it can be seen that the cell sizes collected in the first stage are significantly larger than those in the second stage. The statistical results of the cell number and size are shown in Figure 10. In total, 175 cells were collected in the first stage, 149 of which were larger than 20 μm, 26 were smaller than 20 μm and most were within the range of 20–35 μm. The average cell size was 27.91 μm. In the second stage, 154 cells were collected, of which 54 were larger than 20 μm, 92 were 12–20 μm and 8 were smaller than 12 μm. The average cell size was 18.42 μm, and most cells were within 15–25 μm. The cell size in the second stage was significantly smaller than that in the first stage, consistent with the separation results and the collection results of polystyrene particles. The viability of the collected cells was also analyzed. Among the cells collected in the first stage, the proportion of living cells reached 59%, while only 35% of the cells collected in the second stage were alive. The reason for the large number of dead cells in the second stage may be that the dead cells cannot be pushed away under the effect of nDEP, and they are then deformed and enter the second stage through the microfilter under the effect of the fluid dynamics. However, the degree of deformation is too limited to let the dead cells pass the second stage of filtration, and they then enter the second-stage collection area. Overall, in the first stage, large size and good activity *Haematococcus pluvialis* were collected, while, in the second stage, a small number of large size but poor activity *Haematococcus pluvialis* and some *Bracteacoccus engadinensis* were collected.

## 4. Conclusions

To better solve the blockage problem of the traditional microstructure filtration method, increase the throughput and extend the working life of the chip, we improved the separation microstructure by tilting the micropost array at a certain angle to form a cell track. Compared with previous microfluidic chip [27], the inclined separation micropost array with an optimized angle was integrated to induce cell track. Particles or cells would enter the collection area under micropost array railing, which avoids the potential risk of particles/cells gathered near the filter holes. The effects of the electrode distance and inclination angle of the micropost array on the particle collection efficiency were investigated. Based on the optimized microfluidic chip structure, the proportion of 37-μm particles in the collection area was 85%, and 16.3-μm particles were collected with 89% efficiency, which indicates that the chip has a good separation effect and a high collection efficiency. Additionally, algal cells were collected on the optimized microchip. In the first stage, large size and good activity *Haematococcus pluvialis* were collected, while, in the second stage, a small number of large sizes but poor activity *Haematococcus pluvialis* and some *Bracteacoccus* were collected. The chip also has good separation and collection effects on biological samples, which lays a foundation for subsequent research and application of separation based on microstructure filtration.

## Supplementary Materials

The following are available online at https://www.mdpi.com/2072-666X/11/12/1037/s1, Figure S1: Cells under the microscope: (A) bright field; (B) green light; (C) blue light; and (D) ultraviolet light, Video S1: Particle collection in the first stage, Video S2: Particle collection in the second stage.
