# Supplementary figures and images for: A Continuous Cell Separation and Collection Approach on a Microfilter and Negative Dielectrophoresis Combined Chip
Source: Micromachines (Basel). 2020 Nov 26;11(12):1037. doi: 10.3390/mi11121037 (PMC7759882; doi:10.3390/mi11121037)

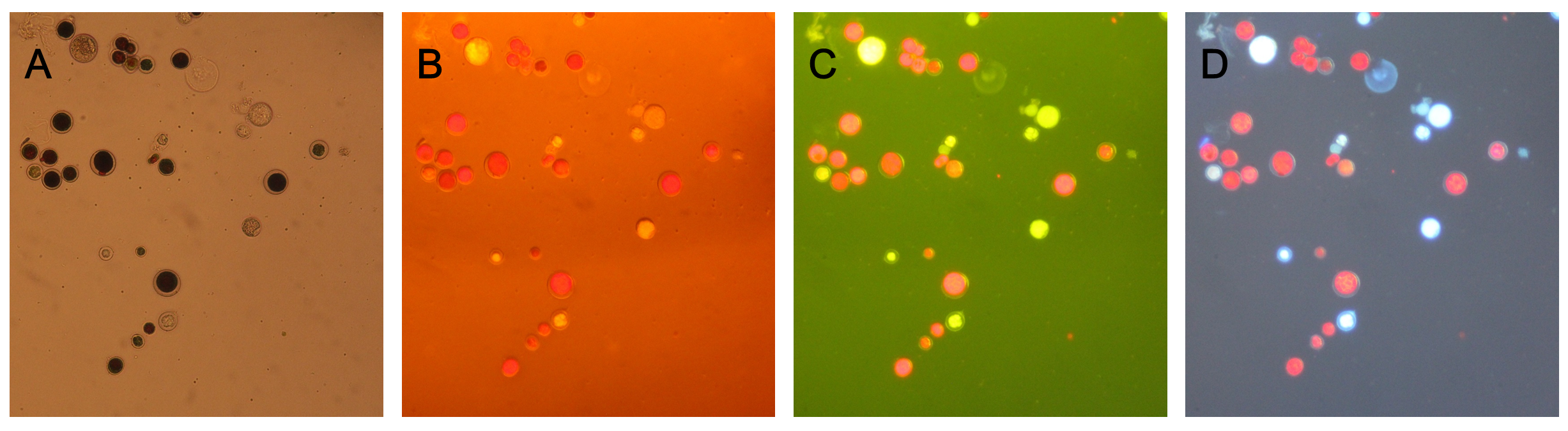

Supplement: Supplementary file 1 [file micromachines-11-01037-s001.zip › SP1.tif]
